# Comparing self-reported sleep quality and wearable-derived sleep metrics in middle-aged and older adults with chronic pain: a psychometric study

**DOI:** 10.3389/fpain.2025.1704377

**Published:** 2025-12-03

**Authors:** Soamy Montesino-Goicolea, Pedro Antonio Valdes-Hernandez, Olga Nin, Cameron Smith, Eric C. Porges, Yenisel Cruz-Almeida

**Affiliations:** 1 Pain Research & Intervention Center of Excellence, University of Florida, Gainesville, FL, United States; 2 Department of Community Dentistry & Behavioral Science, College of Dentistry, University of Florida, Gainesville, FL, United States; 3 Center for Cognitive Aging & Memory, McKnight Brain Foundation, University of Florida, Gainesville, FL, United States; 4 Department of Health Outcomes and Biomedical Informatics (HOBI), College of Medicine, University of Florida, Gainesville, FL, United States; 5 Department of Anesthesiology, College of Medicine, University of Florida, Gainesville, FL, United States; 6 Department of Clinical and Health Psychology College of Public Health and Health Professions, University of Florida, Gainesville, FL, United States; 7 Institute on Aging, University of Florida, Gainesville, FL, United States

**Keywords:** Oura ring, subjective sleep, objective sleep, clinical pain, experimental pain

## Abstract

**Objectives:**

Our primary aim is to evaluate the agreement between subjective and objective methods of measuring sleep quality in people with musculoskeletal pain and poor sleep. Our secondary aim is to explore the relationship between subjective and objective sleep quality in people with clinical and experimental pain, as well as its impact on function.

**Methods:**

Participants with musculoskeletal pain (intensity >5/10 most days over the preceding 3 months) and poor sleep [Pittsburgh Sleep Quality Index (PSQI) total > 5] (*n* = 33) completed the PSQI and wore a ring that characterizes sleep stages (i.e., Oura© ring). Responses to the PSQI over the preceding month were compared to the corresponding averaged Oura measures using zero-order correlations (primary aim). Partial Pearson correlations were used to assess sleep–pain relationships (second aim), controlling for age and sex. Statistical significance was set at *α* <0.05 with Bonferroni correction.

**Results:**

PSQI responses for total bedtime (*p* < 0.0005), sleep duration (*p* < 0.0005), and the PSQI duration component (*p* < 0.003) significantly correlated with their Oura-derived equivalents. In contrast, Oura measures of sleep latency, efficiency, and disturbances showed no alignment with PSQI metrics. The PSQI total score and its sleep latency component were significantly associated with pain measures, including Western Ontario and McMaster Universities Osteoarthritis Index—pain (*p* = 0.022; latency *p* = 0.009), McGill Pain Questionnaire (total *p* = 0.026; latency *p* = 0.008; neuropathic *p* = 0.026, latency *p* = 0.011; continuous *p* = 0.026; intermittent *p* = 0.026, latency *p* = 0.008; affective—latency *p* = 0.008), and Graded Chronic Pain Scale pain intensity (*p* = 0.026; latency *p* = 0.012) as well as interference (latency *p* = 0.008). By contrast, Oura-derived sleep measures showed no significant associations with pain, except for sleep latency, which correlated with conditioned pain modulation (*p* = 0.049). All *p*-values are Bonferroni-corrected.

**Conclusions:**

This preliminary study provides valuable insights into the complementary roles of subjective and objective sleep assessments in older adults with chronic pain. The findings underscore the importance of integrating both approaches to refine sleep evaluation in individuals with musculoskeletal pain. Future research should further examine the feasibility and clinical utility of combining subjective and objective assessments to enhance understanding of sleep- and pain-related health outcomes.

## Introduction

Sleep quality and pain are two critical aspects of health that significantly influence the wellbeing and daily functioning of middle-aged and older adults (1). Chronic pain, prevalent in aging populations, is closely linked with sleep disturbances in a cycle where each exacerbates the other (2). Poor sleep heightens pain sensitivity, while chronic pain disrupts sleep architecture, reducing sleep quality and complicating pain management (1, 3–5). Consequently, accurately assessing sleep quality in the aging population is essential for developing interventions to mitigate both pain and sleep disturbances, thereby improving quality of life (6).

The Pittsburgh Sleep Quality Index (PSQI) (7) is a widely recognized and validated gold standard for assessing subjective self-reported sleep quality, known for its broad applicability across clinical and non-clinical populations and its frequent use as a reference measure in psychometric research (8). It assesses various aspects of sleep, including duration, latency, disturbances, and overall quality over the past month. While valuable in capturing individual perceptions of sleep, the PSQI is subject to recall bias and personal interpretation, which can lead to discrepancies between reported and actual sleep experiences (9, 10). These limitations may be particularly relevant in middle-aged and older adults, where age-related declines in memory and executive function can exacerbate inaccuracies in retrospective self-reporting (11, 12).

Wearable technology has made objective measures of sleep quality more accessible. Devices like the Oura ring (Oura©) use infrared sensors and accelerometers to track sleep stages, providing a comprehensive view of sleep patterns (13, 14), measuring parameters like sleep latency, total sleep duration, sleep efficiency, and heart rate variability (15). Moreover, they are sensitive to aspects of sleep often overlooked or inaccurately reported in self-assessments, such as micro-arousals and subtle disruptions (13, 14). The non-intrusive design and ease of use of the Oura ring make it particularly suited for middle-aged and older adults—a population in which chronic pain is more prevalent—as it promotes compliance over extended periods.

Subjective and objective measures both aim to capture the same construct (i.e., sleep quality); however, discrepancies may arise among individuals with chronic pain. Cognitive-affective processes such as hypervigilance, catastrophizing, and distorted time perception can bias the recall and perception of sleep, influencing metrics like latency, efficiency, and night-to-night variability (1, 16). Consequently, subjective reports may diverge substantially from physiological measurements [e.g., PSQI vs. polysomnography (PSG) or actigraphy (7)]. Moreover, chronic pain often produces fragmented and irregular sleep that is difficult to reconstruct retrospectively, particularly when using self-reporting tools such as the PSQI. Recognizing these divergences is essential for understanding perceptual and cognitive biases in pain-related sleep disturbances and for advancing multimodal assessment strategies that incorporate both subjective experience and objective physiology.

This study primarily aims to bridge the gap between subjective and objective sleep assessments in middle-aged and older adults with musculoskeletal pain by comparing PSQI responses and components with their equivalents estimated from Oura ring data, where applicable. We hypothesize that PSQI measures would significantly and positively correlate with their Oura-based equivalents. The expectation of a positive correlation is based on the rationale that, by design, our Oura equivalents were tailored to mirror PSQI metrics. Only one prior study has reported significant correlations between PSQI and Oura data (17)—PSQI total score significantly correlated with Oura's proprietary Sleep Duration and Efficiency—without tailoring the Oura data to align with PSQI metrics.

In addition, we explored the relationship between sleep and pain in our sample. We hypothesized that subjective sleep-related measures—derived from the PSQI and other questionnaires assessing the impact of sleep quality on function (e.g., daytime sleepiness, daily living activities, and impairment)—would significantly correlate with clinical or experimental pain. Furthermore, we hypothesized that Oura-derived objective sleep quality measures would also significantly correlate with clinical or experimental pain.

Taken together, these aims position the present study to evaluate the utility of integrating subjective and objective sleep assessments in adults with musculoskeletal pain. Establishing the extent to which these measures converge or diverge may inform the development of multimodal strategies to more accurately characterize sleep and its association with pain.

## Participants and Methods

### Ethics statement

This study was approved by the University of Florida (UF) Institutional Review Board and carried out in accordance with the Declaration of Helsinki. Participants provided verbal and written informed consent.

### Participants

This secondary analysis draws on a double-blind, placebo-controlled, parallel-group pilot randomized clinical trial that examined the feasibility of a 4-week regimen of oral γ-aminobutyric acid (GABA) intake (18). The sample comprised 33 middle-aged to older adults recruited between March 2021 and February 2023. Eligibility for the parent study was determined via telephone screening. A detailed description of the screening, inclusion, and exclusion criteria of the parent study has been reported previously (18). In brief, adults aged ≥45 years who owned a smartphone, reported at least moderate pain intensity (>5/10) on most days during the preceding 3 months, and had poor sleep quality (PSQI-Total > 5) were considered for participation. Exclusion criteria included the inability to consent, pregnancy, cognitive impairment, psychiatric and neurological conditions (e.g., schizophrenia, major depressive disorder, bipolar disorder, Alzheimer's disease, Parkinson's disease, epilepsy, and other intracerebral pathologies), mental health–related hospitalization in the past year, serious systemic disorders, arterial hypotension, digestive tract diseases, recent major surgery, chronic or current use of certain medications (including sleep medications), history of alcohol or drug abuse, current cancer diagnosis (unless in remission for ≥2 years), allergies or sensitivity to GABA/placebo or their ingredients, and MRI contraindications. Table 1 summarizes the demographic characteristics of the sample.

**Table 1:** Sample characterization.

Variable	Statistic
Age, mean (SD), years	68 (7.40)
Sex	
Male	7 (21.2%)
Female	26 (78.8%)
Race	
Caucasian	29 (87.9%)
African American	3 (9.1%)
Hispanic	1 (3.0%)
Education level	
High school	7 (21.2%)
Two-year college	8 (24.2%)
Four-year college	8 (24.2%)
Master's degree	7 (21.2%)
Doctorate	3 (9.1%)
Marital status	
Married	19 (57.6%)
Other	14 (42.4%)

### Procedures

During the first visit (baseline visit), participants provided informed consent and underwent a general health assessment. Clinical pain, experimental pain, and subjective sleep (e.g., PSQI) were also assessed. At the end of the visit, participants received an Oura© ring Generation 2 (Gen2), a smart device used primarily to monitor sleep. Participants were instructed to wear the ring continuously at home every night during sleep hours. They were also instructed to open the Oura mobile application each morning to enable the transfer, analysis, and upload of the ring's data for subsequent access. After at least 1 month, participants returned for a follow-up visit to repeat the same assessments conducted during the baseline visit. Detailed information about these and other data can be found in the parent project's publication (18).

### Measures

#### Subjective sleep measure: the PSQI

The PSQI is a well-validated instrument used to assess sleep quality over a month using seven domains (7): sleep quality, sleep latency, sleep duration, habitual sleep efficiency (ratio of total sleep time to bedtime), sleep disturbances, use of sleep-promoting medication, and daytime dysfunction. Each domain is rated on a scale from 0 to 3 (negative extreme). The sum of all domains yields the PSQI total score, ranging from 0 to 21, with a higher score indicating worse sleep quality.

#### Subjective measures of the impact of sleep quality on function

*The Epworth Sleepiness Scale (ESS)* (19): This is a self-administered questionnaire for assessing daytime sleepiness. Individuals rate their propensity to fall asleep in eight distinct situations, including while reading, watching TV, and sitting inactively in a public place. Each situation is scored on a scale from 0 (no chance of dozing) to 3 (high chance of dozing). Their sum is the total score, ranging from 0 to 24, with a score of 10 or more considered indicative of excessive daytime sleepiness.

*The Functional Outcomes of Sleep Questionnaire (FOSQ-10)* (20): This self-administered instrument evaluates the impact of disorders of excessive sleepiness on activities of daily living. It has 10 questions assessing the ability to perform daily activities when feeling tired or sleepy. These activities include concentrating, remembering things, finishing a meal, working on a hobby, working around the house, operating a motor vehicle for short and long distances, driving or taking public transportation, and taking care of financial affairs and paperwork related to employed or volunteer work. Questions are scored on a scale from 1 (yes, extreme difficulty) to 4 (no difficulty). The total score is the sum of the scores, with higher scores indicating better functional status.

*Patient-Reported Outcomes Measurement Information System (Short Form) Sleep-Related Impairment (SF PROMIS-SRI)* (21). This instrument assesses self-reported perception of alertness, sleepiness, and tiredness during usual waking hours and the perceived functional impairments during wakefulness associated with sleep problems and impaired alertness, over 7 days. Raw scores are converted to T-scores (*PROMIS-SRI T-Score*) using the US population as a reference (mean/standard deviation = 50/10), with higher T-scores indicating greater sleep impairment.

#### Objective sleep data: the Oura© ring Gen2

The Oura© ring was selected for its compact design, long battery life, and capacity to continuously collect biometric data in real time, making it especially suitable for use by older adults. The device collects physiological data via integrated sensors—infrared photoplethysmography, temperature, and accelerometry—and processes this information using the proprietary Oura Staging Algorithm to estimate sleep stages (light, deep, and rapid eye movement (REM)).

In addition to sleep staging, the device generates several daily metrics on a scale of 0–100, including the *sleep score*, *sleep efficiency score*, *restfulness score*, *sleep latency score*, and *sleep timing score*, along with estimations of *total bedtime*, *total sleep time*, and sleep latency in minutes and hours.

In this study, the Oura-derived scores and estimates were used to construct PSQI-equivalent variables for the primary aim and averaged across all available days of usage for secondary analyses.

#### Oura-derived PSQI equivalents

To compare subjective and objective sleep (our primary aim), we first calculated Oura-based equivalents for the PSQI responses and components where possible. Since the PSQI assesses sleep over the preceding 30 days, we used Oura scores and estimates recorded during the 30 days preceding the administration of the PSQI questionnaire to derive these equivalents. However, based on the available Oura data, only a subset of PSQI responses and components could be identified or calculated. The details of these equivalents are provided in Table 2.

**Table 2:** Details of the calculation of Oura's equivalent items of the PSQI, used to test hypothesis 1.

PSQI item	Description of related question or component	Value (variable type) estimation	Oura equivalent
PSQI1	“During the past month, what time have you usually gone to bed at night?”	Hour (hour)	Not available
PSQI3	“During the past month, what time have you usually got up in the morning?”	Hour (hour)	Not available
Total bedtime	PSQI3 − PSQI1	Hours (continuous)	1-month average of total bedtime
PSQI2	“During the past month, how long (in minutes) does it usually take you to fall asleep each night?”	Minutes (continuous)	1-month average of sleep latency score
PSQI4	“During the past month, how many hours of actual sleep did you get at night? (This may be different than the number of hours you spent in bed)”	Hours (continuous)	1-month average of total sleep time
PSQI5a	“During the past month, how often have you had trouble sleeping because you: a) Cannot get to sleep within 30 min)”	Single choice (ordinal, values shown below): - Not during the past month (0) - Less than once a week (1) - Once or twice a week (2) - Three or more times a week (3)	Occurrences of sleep latency score >30 min, then categorizing accordingly
C1	Severity of poor overall sleep quality. This is PSQI9: “During the past month, how would you rate your sleep quality overall?”	Single choice (ordinal, values shown below): - Very good (0) - Fairly good (1) - Fairly bad (2) - Very bad (3)	Sleep score (0–100) categorized as follows: [0, 55) → 3 [55, 70) → 2 [70, 85) → 1 [85, 100] → 0
C2	Severity of longer sleep latency	0, 1, 2, 3 (ordinal) Using PSQI2 and PSQI5a according to the PSQI scoring (7)	Using the Oura equivalents, according to the PSQI scoring (7) (see cells to the left)
C3	Severity of shorter sleep duration	0, 1, 2, 3 (ordinal) Using PSQI4 according to the PSQI scoring (7)	
C4	Severity of lower efficiency	0, 1, 2, 3 (ordinal) Using total bedtime and PSQI4 according to the PSQI scoring (7)	
C5	Severity of frequent sleep disturbances	0, 1, 2, 3 (ordinal) Using PSQI5b–PSQI5j according to the PSQI scoring (7)	Since Oura equivalents of PSQI5b-PSQIj are not available, we used, as a proxy, the restfulness score (0–100) categorized as follows: [0, 70) → 3 [70, 85) → 2 [85, 100) → 1 100 → 0

PSQI[X], answer to question X of the PSQI. Higher component (C) value scores mean worse sleep quality. Averages were calculated excluding any missing entries.

Based on the assumption that participants recall recent sleep more accurately than distant sleep, we examined the potential impact of recall bias on PSQI responses. Specifically, we recalculated the Oura-derived sleep metrics (as shown in Table 2) using data from shorter, more recent time windows—25, 20, 15, and 10 days prior to PSQI administration. These windows were selected to model increasing levels of recall reliability. This approach allowed us to assess whether correlations between subjective (PSQI) and objective (Oura) sleep measures varied as a function of recall proximity.

We note that the Oura Sleep Score is a proprietary composite index and, as such, is not a direct analog of the PSQI-Total. Nevertheless, both aim to capture the same underlying construct—overall sleep quality (13, 15)—which justifies their comparison. A similar rationale was applied to the comparison between the Oura Restfulness Score and PSQI Component 5 (PSQI C5: Sleep Disturbances).

#### Clinical pain measures

*Western Ontario and McMaster Universities Osteoarthritis Index—Pain (WOMAC-pain)*: This subscale assesses pain experienced in the lower limbs during various activities (3). Items are rated on a 5-point scale ranging from 0 to 20, with higher scores indicating greater pain during activities.

*Short-Form McGill Pain Questionnaire-Revised (SF-MPQ-2)*: This questionnaire is used to measure the quality and the intensity of current pain (22). It comprises the subscales of SF-MPQ-2-Continuous pain, SF-MPQ-2-Intermittent pain, SF-MPQ-2-Neuropathic pain, and SF-MPQ-2-Affective experiences. Each of the 22 pain descriptors is rated on a scale of 0 (“no pain”) to 10 (“worst pain ever”) based on the preceding week, and an SF-MPQ-2-Total sum score is calculated for each subscale.

*Graded Chronic Pain Scale (GCPS)*: This seven-item questionnaire provides two subscales: pain intensity (GCPS-Intensity) and pain interference (GCPS-Interference) over the past 6 months (23). For the GCPS-Intensity, participants rate their current, average, and worst pain on a 0 (“no pain”) to 10 (“pain as bad as could be”) numerical rating scale. These are averaged and multiplied by 10 to yield a 0–100 score. Likewise, for the GCPS-Interference, participants rate on a 0 (“no inference”) to 10 (“unable to carry out activities”) scale the extent to which pain has interfered with daily activities, recreational/social/family activities, and ability to work on average, over the past 6 months. These ratings are also averaged and multiplied by 10.

#### Experimental pain measures

Quantitative sensory testing (QST) was conducted at standardized anatomical sites and at a region identified as painful by the participant. Testing employed the TSA-II Neurosensory Analyzer and accompanying software (Medoc Ltd., Ramat Yishai, Israel), a TCS system (QST-Lab, https://www.qst-lab.eu/, France), and the AlgoMed computerized algometer (Medoc Ltd., https://www.medoc-web.com, Israel). More details can be found in our earlier publication (18).

*Vibration detection threshold*: Using a handheld VSA-3000 circular probe (contact tip = 1.22 cm^2^) from the TSA-II Neurosensory Analyzer with accompanying software (Medoc Ltd., Ramat Yishai, Israel), a 100-Hz vibratory stimulus (ramping from 0 μm at a 0.5 μm/s rate) was delivered to the thenar as well as the painful site until the participant perceived the vibratory sensation. The average threshold across three trials, every 10 s, was calculated.

*Pressure pain threshold*: Using the AlgoMed computerized algometer (with a 10-mm rubber tip), pressure (kg/s rate) was applied to the right quadriceps muscle and a painful site—order randomized and counterbalanced—until the participant indicated that the sensation “first became painful.” The average threshold was calculated across three trials.

*Mechanical temporal summation*: A pressure of 300 g was applied to the thenar and a painful site using a nylon monofilament (TouchTest Sensory Evaluator 6.65)—order randomized and counterbalanced. Participants rated their pain after a single contact with the monofilament and after ten consecutive contacts at a 1 contact/s rate. The difference between these ratings was used to evaluate temporal summation of mechanical pain.

*Conditioned pain modulation (CPM)*: CPM was assessed to evaluate the endogenous pain inhibitory function. The test stimulus (TS) consisted of heat pain applied to the left ventral forearm using the QST-Lab equipment with a T08 thermode. The temperature started at 32°C and increased gradually until participants reported a pain intensity of 40/100 on a numerical rating scale and pressed a button that immediately returned the temperature to baseline (32°C). This sequence was repeated three times, and the average temperature at which participants reached the 40/100 pain rating was recorded (denoted as T_pre_). Participants then immersed their right hand in 10°C water (conditioning stimulus, CS) for 60 s. Immediately after, the heat pain procedure on the forearm was repeated three times, and the mean temperature at which the 40/100 pain threshold was reached again was recorded (denoted as T_post_). CPM was defined as (T_pre_ − T_post_)/T_pre_ × 100. Negative CPM values reflect increased pain thresholds (stronger descending inhibition) and positive values indicate reduced inhibition or pain facilitation.

### Statistical analysis

Pearson partial correlations, controlling for age and sex, were used to assess the relationship between continuous variables, and Spearman partial correlations were applied for categorical variables. Pairwise deletion was used to handle missing data, resulting in a variable sample size for each correlation. This approach was chosen to maximize the use of available data and maintain statistical power, as the amount of missingness was minimal and did not follow any systematic pattern.

To test our first hypothesis, we calculated the zero-order correlations between the PSQI items at follow-up and their corresponding Oura metrics from the preceding one-month period of ring usage, as outlined in Table 2. Since the PSQI captures self-reported sleep quality over the preceding month, it aligns temporally with the Oura data collected during the same period, making the follow-up PSQI more appropriate for this analysis than the baseline assessment. A one-tailed significance of 0.05 was applied, after Bonferroni correction for multiple comparisons.

For the second and third hypotheses, each variable of the first (the subjective sleep-related measures) and second columns (the objective sleep-related measures) of Table 3, respectively, was correlated with each variable of the last two columns (the pain measures), controlling by age and sex (i.e., using Pearson partial correlations). A two-tailed statistical significance of 0.05 was applied, after Bonferroni correction for multiple comparisons. All correlations between sleep and pain variables were conducted using pre-intervention (baseline) data, prior to any exposure to GABA or placebo.

**Table 3:** Variables used for the sleep–pain correlations (used for testing hypotheses 2 and 3).

Sleep measures		Pain measures	
Subjective	Generated by Oura	Clinical	Experimental (QST)
PSQI-total score	Sleep score	WOMAC-Total	Vibratory detection threshold-thenar
Epworth total score^a^	Sleep efficiency score	WOMAC-Pain	Vibratory detection threshold-painful site
FOSQ10 total score^a^	Restfulness score	WOMAC-Stiffness	Mechanical temporal summation-thenar
SF PROMIS-SRI T-Score^a^	Sleep latency score	WOMAC-Physical Function	Mechanical temporal summation-painful site
	Sleep timing score	MPQ-Total	Pressure pain threshold-quadriceps
	Total bedtime	MPQ-Continuous	Pressure pain threshold-painful site
	Total sleep time	MPQ-Intermittent	Conditioned pain modulation
	Sleep latency	MPQ-Affective	
		MPQ-Neuropathic	
		GCPS-Intensity	
		GCPS-Interference	

a Subjective measures of the impact of sleep quality on function.

The measures generated by Oura, collected over several days between the baseline and follow-up visits, were averaged for this analysis.

With *n* = 33 and two-tailed tests, the study had ∼80% power to detect medium-to-large correlations. For zero-order correlations (df = 31), the minimum detectable effect (MDE) is *r* ≈ 0.46 at *α* = 0.05 and *r* ≈ 0.55 under a representative Bonferroni adjustment (e.g., *α* ≈ 0.0083 for six comparisons). For partial correlations controlling for age and sex (df = 29), the MDE ranged from *r* ≈ 0.47–0.57 as α tightened from 0.05 to ≈0.0083. These thresholds are consistent with the per-family Bonferroni levels applied in our analysis.

## Results

### Oura ring usage summary

**Figure 1:**
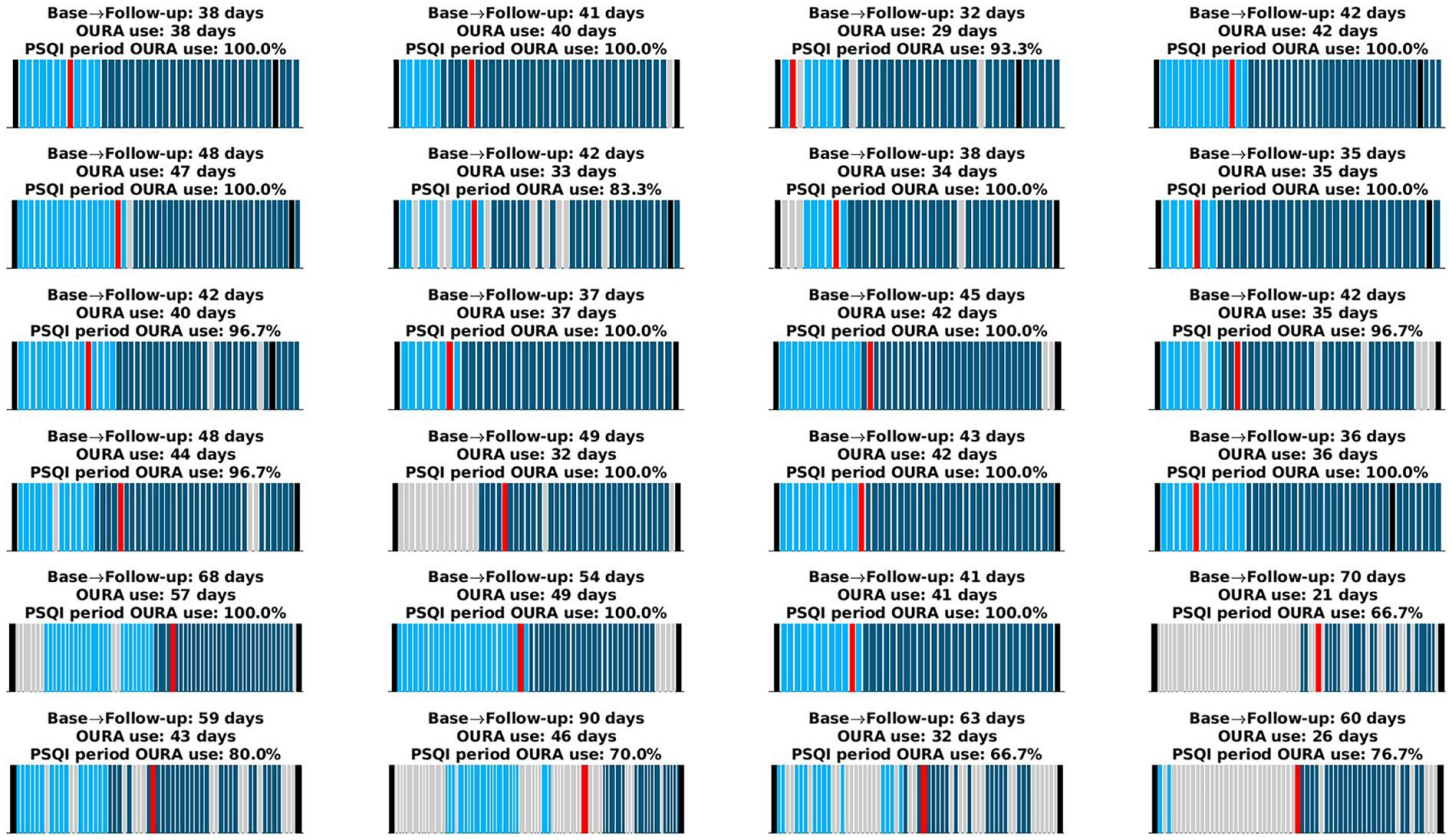
Oura ring usage for each participant. The *x*-axis spans the period from the time participants received the Oura ring until they returned it. Black lines indicate the baseline and follow-up visits. Light cyan bars represent days the Oura ring was worn during the baseline period, and dark cyan bars represent days during the intervention period. Gray bars indicate days the device was not worn. The red bar marks the start of the one-month period preceding the PSQI administration, which occurred during the follow-up visit. Each subplot's title provides (i) the total number of days between the baseline and follow-up visits, (ii) the total number of days the Oura ring was worn (used to compute averages for the second aim), and (iii) the percentage of days the ring was worn within the 1-month period preceding the PSQI administration (used to derive PSQI equivalents for the primary aim). Some participants continued using the ring beyond the follow-up visit; these data were reserved for further analyses.

Within the 1-month period preceding the PSQI administration at follow-up, from which Oura data were used for our primary aim, participants consistently wore the ring between 66.7% and 100% of the days. The mean usage was 92.8%, with a standard deviation of 11.7% and a median of 100%. More generally, across the entire study period, participants wore the Oura ring for 21–57 days, with a mean of 38.4 days, a standard deviation of 7.8 days, and a median of 39 days. These metrics were used to compute the average of Oura's proprietary scores and estimates for our secondary aim. Further details on individual Oura ring usage patterns, including distinctions between the baseline and intervention periods, are presented in Figure 1.

### Comparison between subjective and objective sleep

**Figure 2:**
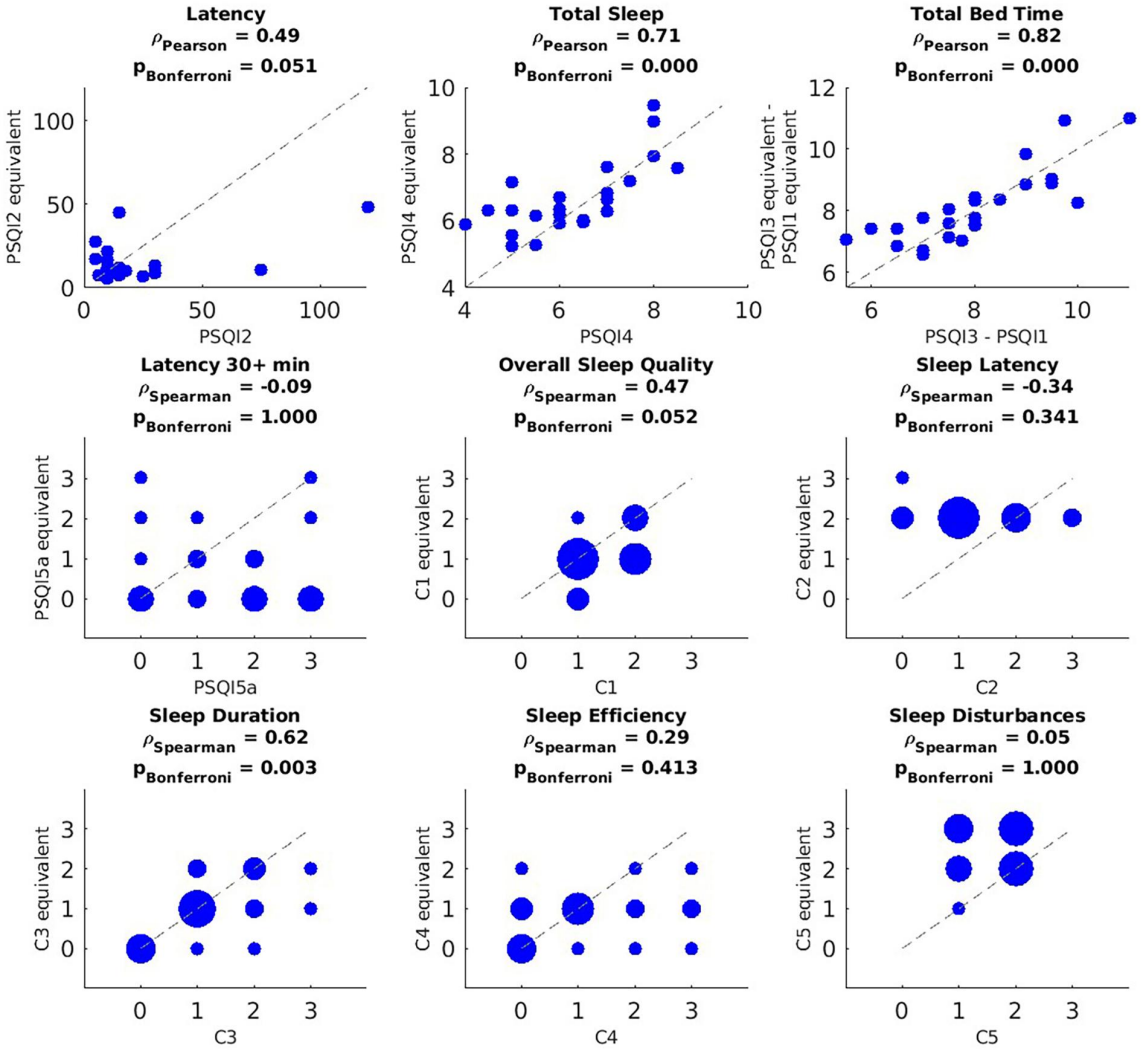
Correlation between PSQI measures and their Oura equivalents based on data recorded during the month preceding the PSQI questionnaire. PSQI question or component (*x*-axis) vs. its Oura-derived equivalent (*y*-axis). For the discrete variables (i.e., PSQI5a, C1–C5), blob size is proportional to the number participants in that value pair (bigger blobs in the *y* = *x* line mean less discrepancy between the subjective measure and its objective equivalent). *P*-values were corrected using Bonferroni across questions and components independently. After pairwise deletion, the sample size used for these correlations was *n* = 25.

The correlation between the PSQI items and their 1-month Oura equivalents, introduced in Table 2, are shown in Figure 2 (*n* = 25 after pairwise deletion). A significant positive Pearson correlation was observed between the PSQI and the Oura equivalents for PSQI2 (*r* = 0.49, *p* = 0.051, though marginally, as this seems to be driven by two outliers), PSQI4 (*r* = 0.71, *p* < 0.001), and total bedtime (PSQI3 − PSQI1; *r* = 0.82, *p* < 0.001) and C3 (severity of longer sleep duration; *r* = 0.62, *p* = 0.003). There was a marginally significant positive correlation between the PSQI's C1 and its equivalent (severity of poor overall sleep quality; *r* = 0.47, *p* = 0.052). This is noteworthy because the correlation becomes significant when accounting for potential recall bias, specifically by limiting the Oura equivalents to any of the most recent 25, 20, 15, or 10 days of ring usage as shown in Supplementary Figures S1–S4. These figures also demonstrate that the remaining significant and non-significant results persist when accounting for potential recall bias.

### Comparison between sleep and pain

Analyses involving sleep–pain associations were restricted to pre-intervention (baseline) data to ensure independence from treatment effects (see Figure 1 for study timeline). With a sample size determined by pairwise deletion, several significant correlations were observed after adjusting for multiple comparisons. PSQI-Total Score exhibited positive correlations with WOMAC-Pain (*r* = 0.53, *p* = 0.022, *n* = 32), MPQ-Total (*r* = 0.50, *p* = 0.026, *n* = 29), MPQ-Neuropathic (*r* = 0.45, *p* = 0.026, *n* = 31), MPQ-Continuous (*r* = 0.48, *p* = 0.026, *n* = 29), MPQ-Intermittent (*r* = 0.46, *p* = 0.026, *n* = 31), and GCPS-Intensity (*r* = 0.45, *p* = 0.026, *n* = 31). In addition, the PSQI Component C2 showed positive correlations with WOMAC-Pain (*r* = 0.51, *p* = 0.009, *n* = 32), MPQ-Total (*r* = 0.58, *p* = 0.008, *n* = 29), MPQ-Neuropathic (*r* = 0.49, *p* = 0.011, *n* = 31), MPQ-Intermittent (*r* = 0.55, *p* = 0.008, *n* = 31), MPQ-Affective (*r* = 0.53, *p* = 0.008, *n* = 32), GCPS-Intensity (*r* = 0.49, *p* = 0.012, *n* = 31), and GCPS-Interference (*r* = 0.53, *p* = 0.008, *n* = 31). Furthermore, the CPM showed a positive correlation with the average of Oura's sleep latency (*r* = 0.626, *p* = 0.049, *n* = 22). However, there were no significant associations between objective sleep measures and clinical pain measures.

## Discussion

Our study primarily aimed to evaluate the relationship between subjective and objective assessments of sleep quality in middle-aged and older adults with chronic musculoskeletal pain and poor sleep. Our findings confirm previous findings that self-reported sleep taps into distinct dimensions that can be complemented and enhanced by objective assessments using wearable technologies.

Consistent with studies using PSG and actigraphy (17, 24–26), we observed correlations between self-reported sleep duration and Oura's sleep duration. This suggests that, despite potential limitations such as recall bias (27), subjective reports provide meaningful insights into sleep duration and bedtime in middle-aged and older adults with chronic pain and poor sleep. In our sample, the strong correlation between PSQI and Oura for bedtime may also reflect structured sleep habits adopted by individuals managing chronic conditions, as part of self-management strategies to improve wellbeing and minimize pain (1, 28).

When using the full 1-month Oura dataset, the PSQI–Oura correlation for overall sleep quality was marginal. However, this correlation became significant when analyses focused on more recent sleep periods, suggesting the influence of recall bias. Discrepancies between subjective and objective sleep quality over longer recall windows may be shaped by physiological (e.g., hyperarousal), psychological (e.g., catastrophizing), and social factors, in line with the biopsychosocial model of pain (29). Chronic pain is known to alter central nervous system processing and emotional states, which can distort the subjective perception of sleep quality (30), exacerbating recall bias. For example, individuals with chronic pain may be more sensitive to minor sleep disruptions or may struggle to separate their pain experience from their perception of sleep quality, even when objective data indicate otherwise. In line with previous research (7), our findings demonstrate that discrepancies between subjective and objective sleep assessments persist even after accounting for recall bias. In particular, aspects such as prolonged sleep latency (e.g., nights exceeding 30 min), efficiency, and disturbances were not captured in the same way by the PSQI as by the Oura ring. Although the PSQI includes items related to these domains, our findings suggest it may underrepresent specific patterns—such as the frequency of prolonged sleep latency or variability in night-to-night disturbances—relative to continuous, objective data. These differences likely reflect limitations of retrospective self-reported measures, which are less sensitive to short-term fluctuations and may overlook features like wake after sleep onset (WASO) or micro-awakenings detectable by wearables.

For instance, while objective measures of WASO were positively associated with the PSQI sleep disturbance component, the PSQI total score showed no correlation with objective sleep parameters (31). Furthermore, chronic pain is associated with a heightened risk of sleep disturbances that may be underestimated through self-reporting alone (30). Moreover, patients with chronic pain and insomnia often overestimate sleep latency compared to PSG (24). Overall, the discrepancies between subjective and objective sleep assessments in our sample emphasize the need for cautious interpretation of self-reported sleep data and highlight the importance of integrating objective measures to achieve a more comprehensive evaluation of sleep quality in middle-aged and older individuals, particularly those living with chronic pain.

Taken together, the findings related to our first hypothesis emphasize the benefits of a multimodal approach to sleep assessment, enabling a more comprehensive understanding of sleep quality by capturing both perceived and physiological aspects. These findings have important implications for clinical practice. Healthcare providers treating patients with chronic pain should consider incorporating objective sleep measurements, particularly in cases where poor sleep quality significantly impacts pain management and overall quality of life. Wearable devices like the Oura ring provide a non-invasive and reliable method for obtaining detailed sleep data, offering clinicians actionable insights to inform personalized treatment strategies.

Shifting focus to our second hypothesis, we found significant positive correlations between self-reported overall sleep quality, as measured with the PSQI-Total Score, and various self-reported measures of pain severity. This finding aligns with the understanding that sleep and pain are closely interconnected and can influence each other (2, 30). Chronic pain can disrupt sleep, leading to poor sleep quality (4, 32–34), while poor sleep can, in turn, exacerbate pain perception and increase sensitivity to painful stimuli (5, 35). The positive correlation observed between the severity of longer sleep latency, assessed with the PSQI, and multiple pain measures suggests that difficulty falling asleep may contribute to—or reflect—greater pain severity in this population. This finding warrants further investigation to understand the specific mechanisms underlying this relationship, as well as its directionality.

With regard to our third hypothesis, no significant associations were found between pain and objective sleep data obtained from the Oura ring. However, given the significant relationship between pain and subjective sleep, this discrepancy invites further exploration of potential factors contributing to the divergence between subjective and objective sleep measures in individuals with chronic musculoskeletal pain. One possibility is that individuals with chronic pain may perceive sleep disturbances as more bothersome due to heightened pain sensitivity, potentially distorting their PSQI reports (36, 37). This distortion could weaken the alignment between subjective reports and the physiological sleep data captured by the Oura ring. While pain itself may be a contributing factor, this remains speculative and warrants further investigation. Future studies with adequate power should examine the moderating effect of pain on the relationship between objective and subjective sleep variables.

Interestingly, we observed a positive correlation between CPM and the 1-month average of Oura's sleep latency. In this paradigm, higher (more positive) CPM values indicate reduced inhibitory efficiency, whereas more negative values reflect stronger descending pain inhibition. Therefore, this finding suggests that participants with longer sleep latency—who take more time to fall asleep—exhibited weaker endogenous pain inhibition, consistent with a potential shift toward pain facilitation observed in chronic pain conditions. This result aligns with prior evidence linking poor sleep quality, hyperarousal, and diminished descending pain control (38, 39). Nevertheless, given the modest sample size and the exploratory nature of this study, further research is warranted to clarify the mechanisms linking sleep latency and endogenous pain modulation (40).

Finally, psychological factors such as anxiety, depression, and stress, commonly comorbid with chronic pain (41, 42), can significantly impact both sleep quality and pain perception (43, 44). Conversely, positive encounters, such as meaningful social interactions or enjoyable daily experiences, may act as a buffer against these negative psychological influences (45). These psychological factors are more likely to be reflected in self-reported measures like the PSQI than in objective sleep data.

### Limitations

The current study includes a very specific population (i.e., middle-aged to older individuals with poor sleep), thereby constraining the generalizability of the findings. Because the sample consisted exclusively of individuals with chronic pain and poor sleep, the absence of a pain-free control group further limits the generalizability of our results. Future studies should include healthy or non-painful comparison groups to determine whether the discrepancy between subjective and objective sleep assessments reflects pain-related pathology or broader age-related phenomena.

This report should be regarded as preliminary due to its small sample size, which limits the statistical power typically required for psychometric studies and may increase the risk of type II errors. More importantly, a small sample size also reduces the likelihood that statistically significant findings reflect true effects. Because this likelihood is inversely proportional to the type I error, more stringent statistical thresholds are necessary to protect against spurious positive results. Consequently, some of our results with corrected *P*-values near 0.05 would be interpreted as only marginally significant. However, this concern is partially mitigated by the richness of the objective data collected across multiple days using the Oura ring, which increases measurement stability, and by the conservative use of multiple comparison corrections. Nevertheless, replication in larger and more diverse samples is essential to confirm and extend these findings.

In addition, reliance on a single wearable device restricts the applicability of results to other technologies that may use different algorithms or exhibit varying sensitivities. It is also important to acknowledge the inherent limitations of wearable technology in capturing the full complexity of subjective sleep experiences. Specifically, the Oura Sleep Score is derived from proprietary algorithms and is not a direct analog of the PSQI-Total, which reflects a combination of self-reported components based on standardized sleep-related questions. Nevertheless, both measures aim to capture the same overarching construct—overall sleep quality. This conceptual overlap justifies their comparison and provides meaningful insight into the relationship between subjective perceptions and sensor-derived estimates of sleep. We applied the same rationale to compare the Oura Restfulness Score with PSQI Component 5 (C5). However, we acknowledge that the restfulness score does not directly reflect the specific causes of sleep disturbances queried in C5 (e.g., nighttime awakenings, bathroom use, and physical discomfort). Instead, it was used as a proxy, based on its intended reflection of uninterrupted, high-quality sleep. While this provides only a partial alignment, it represented the most relevant available metric for exploratory comparison.

Future studies should include larger, more diverse populations, incorporate a broader range of wearable technologies, and adopt longitudinal designs to better understand how subjective and objective sleep measures interact and evolve across different contexts.

## Conclusions

These exploratory findings underscore the value of integrating subjective and objective sleep assessments in individuals with chronic musculoskeletal pain. The PSQI remains a clinically meaningful tool, particularly given its strong associations with pain, but it may not fully capture certain physiological patterns detectable with wearable technology. Devices like the Oura ring provide complementary insights, especially for aspects such as variability or micro-awakenings that are difficult to assess retrospectively. Together, these approaches offer a more complete evaluation of sleep, supporting both research and clinical strategies to improve health outcomes in aging populations (1, 46).

## Data Availability

The raw data supporting the conclusions of this article will be made available by the authors, without undue reservation.
