# Supplementary material for: Comparing self-reported sleep quality and wearable-derived sleep metrics in middle-aged and older adults with chronic pain: a psychometric study
Source: Front Pain Res (Lausanne). 2025 Dec 3;6:1704377. doi: 10.3389/fpain.2025.1704377 (PMC12708585; doi:10.3389/fpain.2025.1704377)
Supplement: Supplementary file 1 [file Table1.docx]

Supplemental Materials


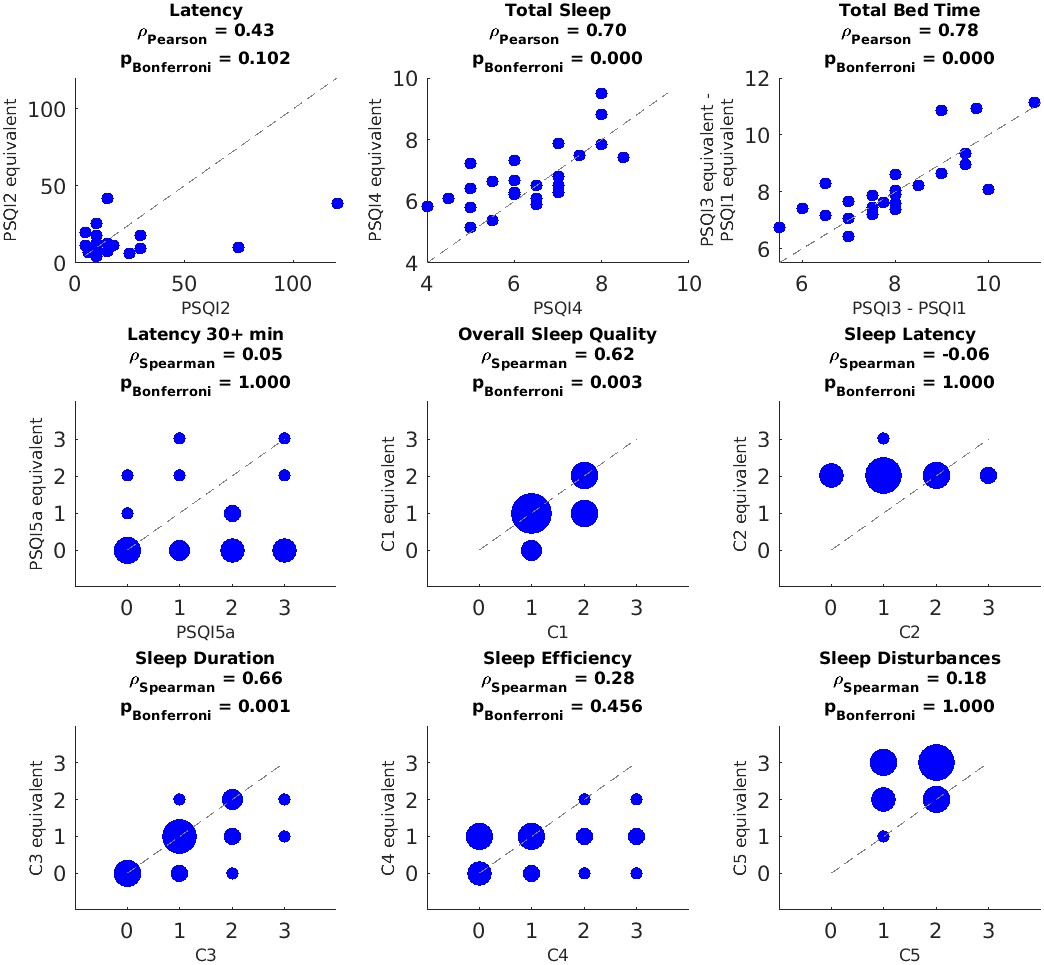


Figure S1 **Correlation Between PSQI Measures and their Oura Equivalents Based on Data Recorded During the Last 10 days Preceding the PSQI Questionnaire**. PSQI question or component (x axis) versus their Oura-derived equivalents (y axis). For the discrete variables (i.e., PSQI5a, C1-C5), blob size is proportional to the number participants in that value pair (bigger blobs in the y=x line means less discrepancy between the subjective measure and its objective equivalent). P-values were corrected using Bonferroni across questions and components independently.


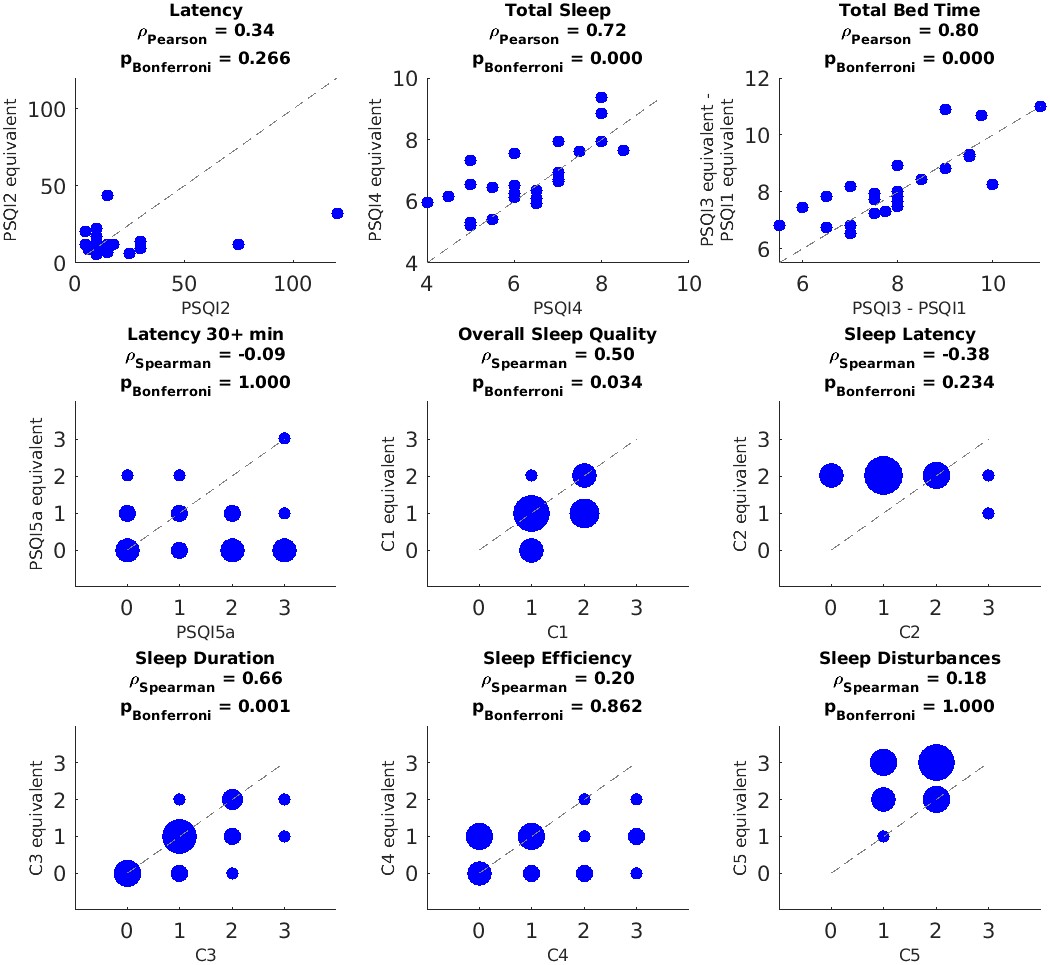


Figure S2 **Correlation Between PSQI Measures and their Oura Equivalents Based on Data Recorded During the Last 15 days Preceding the PSQI Questionnaire**. PSQI question or component (x axis) versus their Oura-derived equivalents (y axis). For the discrete variables (i.e., PSQI5a, C1-C5), blob size is proportional to the number participants in that value pair (bigger blobs in the y=x line means less discrepancy between the subjective measure and its objective equivalent). P-values were corrected using Bonferroni across questions and components independently.
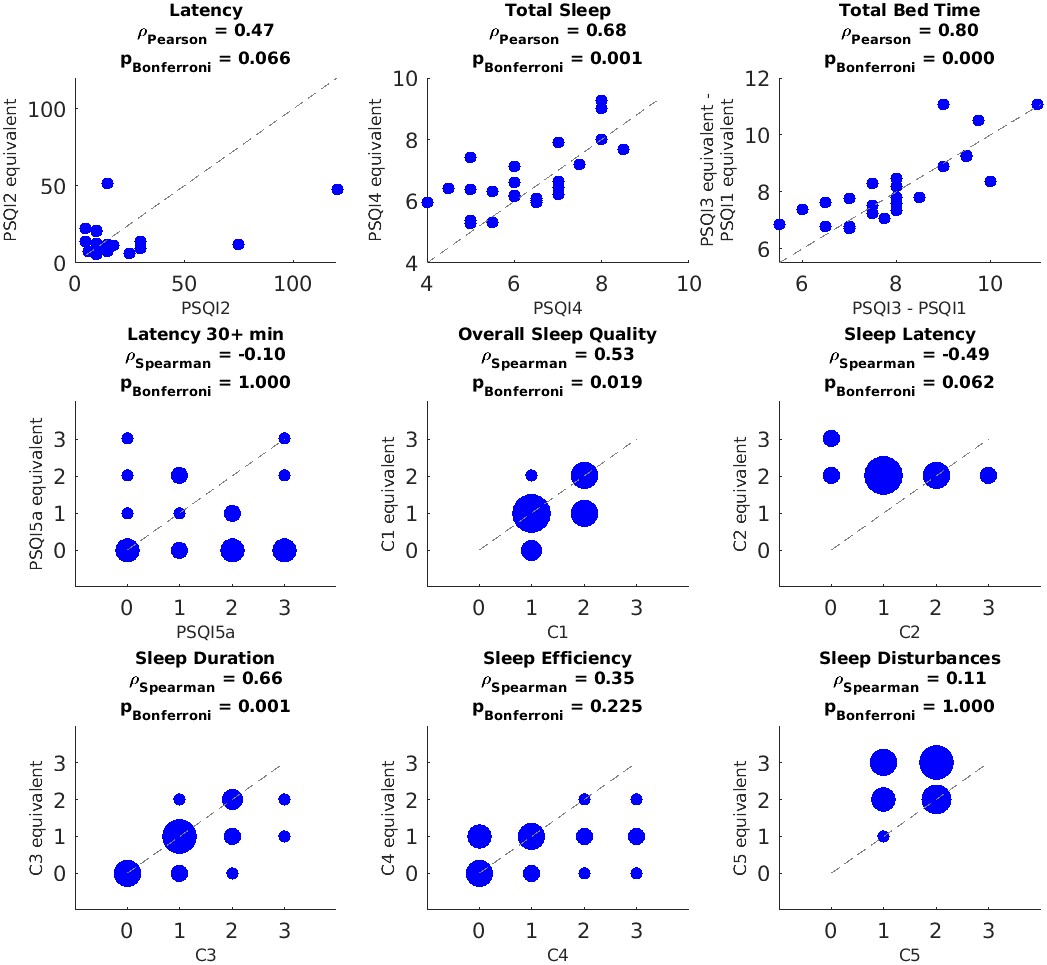


Figure S3 **Correlation Between PSQI Measures and their Oura Equivalents Based on Data Recorded During the Last 20 days Preceding the PSQI Questionnaire**. PSQI question or component (x axis) versus their Oura-derived equivalents (y axis). For the discrete variables (i.e., PSQI5a, C1-C5), blob size is proportional to the number participants in that value pair (bigger blobs in the y=x line means less discrepancy between the subjective measure and its objective equivalent). P-values were corrected using Bonferroni across questions and components independently.


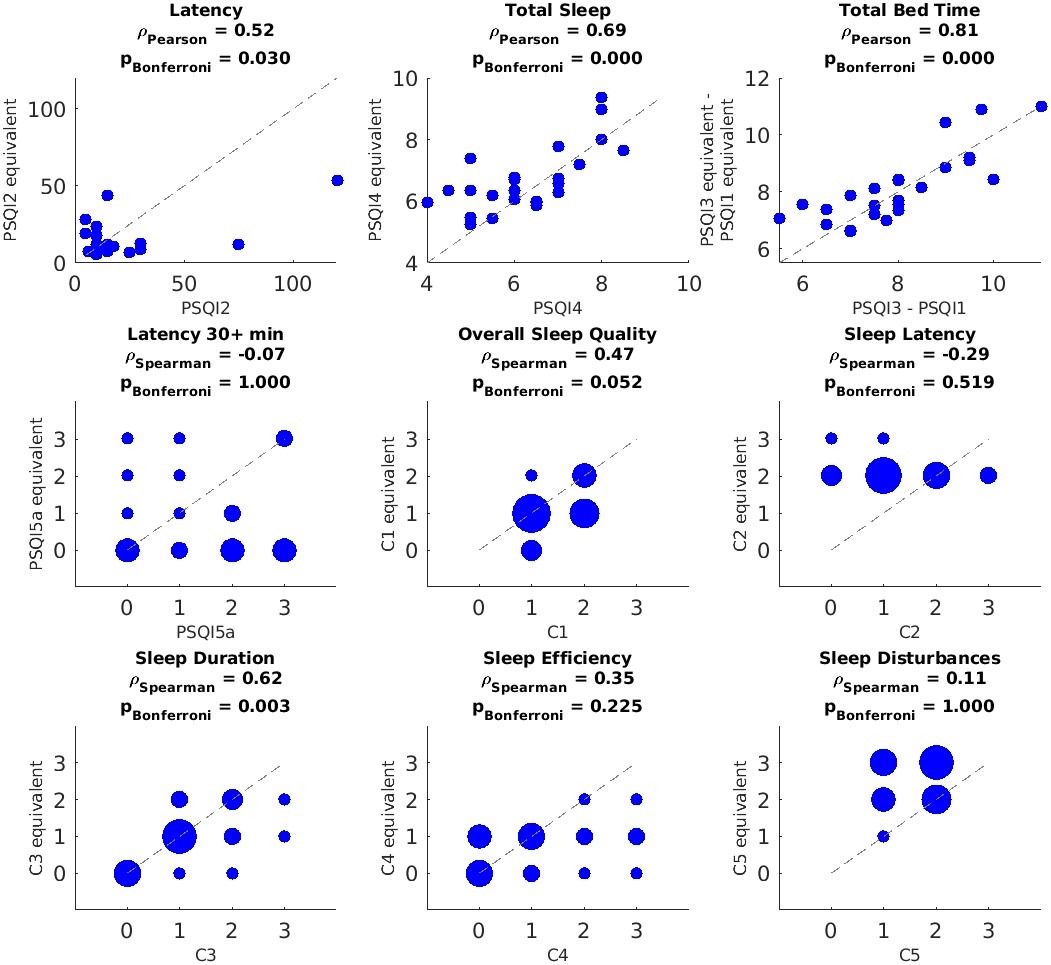


Figure S4 **Correlation Between PSQI Measures and their Oura Equivalents Based on Data Recorded During the Last 25 days Preceding the PSQI Questionnaire**. PSQI question or component (x axis) versus their Oura-derived equivalents (y axis). For the discrete variables (i.e., PSQI5a, C1-C5), blob size is proportional to the number participants in that value pair (bigger blobs in the y=x line means less discrepancy between the subjective measure and its objective equivalent). P-values were corrected using Bonferroni across questions and components independently.
